# Assessing fidelity to complex interventions: the icons experience

**DOI:** 10.1186/1745-6215-14-S1-P4

**Published:** 2013-11-29

**Authors:** Brigit Chesworth, Michael Leathley, Lois Thomas, Denise Forshaw, Chris Sutton, Bev French, Chris Burton, David Britt, Brenda Roe, Francine Cheater, Caroline Watkins

**Affiliations:** 1 University of Central Lancashire, Preston, UK; 2 Edge Hill University, Ormskirk, UK; 3 Bangor University, Bangor, UK; 4 University of East Anglia, Norwich, UK

## Background

Assessing fidelity to complex healthcare interventions in clinical trials is a challenging area. ‘ICONS' is a cluster randomised controlled feasibility trial of a systematic voiding programme (SVP), incorporating bladder training and prompted voiding, to promote post-stroke continence. Here we describe feasibility of one aspect of fidelity assessment: the day-to-day implementation of the SVP through analysis of clinical logs.

## Methods

Nurses completed clinical logs daily, which included documenting: the toileting interval, proposed toileting times and times toileted. Clinical logs were sampled across trial sites. The original intention was to assess fidelity by exploring the degree of concordance between proposed times and times toileted. Initial analysis revealed the unfeasibility of this method due to documentation errors in toileting intervals and proposed times. Consequently, the planned method was changed to identification of key ‘quality indicators' (QIs) for documentation of practice.

## Results

The need to revise the method of measurement demonstrates the difficulty in assessing fidelity. Assessment of clinical logs revealed low levels of adherence to key quality indicators. However, it is unclear whether this indicates poor fidelity or an imprecise method of fidelity assessment.

## Conclusion

This study highlights challenges of assessing fidelity to complex interventions. Lessons learned will inform the measurement of fidelity in a future trial. Researchers should be aware that the practical implementation of complex healthcare interventions may not be exactly as intended. For ICONS, clinical logs constituted a proxy measure of day-to-day fidelity to the intervention: identification of alternative methods could be considered.

